# The new World Health Organization recommendation on the 2-dose measles vaccine schedule and the way forward in African Region

**DOI:** 10.11604/pamj.supp.2017.27.3.11611

**Published:** 2017-06-21

**Authors:** Robin Julian Biellik, Robert Davis

**Affiliations:** 1 WHO African Regional Measles-Rubella Technical Advisory Group, Geneva; 2 American Red Cross, Nairobi, Kenya

**Keywords:** Immunization, measles, Africa

## Abstract

The new W.H.O. recommendation, which drops the coverage criterion for adoption of the 2-dose measles vaccine schedule, makes some African countries eligible for the 2-dose schedule which were previously ineligible. We look at the implications of the new recommendation for Ethiopia and Nigeria, the two largest African countries which are eligible under the new recommendation.

## Editorial

Two dose measles vaccination regimes need to be seen in light of the move from single dose to two dose schedules at the global and the national level. Two dose regimes started in the developed world, and have spread, though not quickly, to the developing world. “During 2000-2015, the number of countries providing MCV2 nationally through routine immunization services increased from 97 (51%) to 160 (82%), with 6 countries (Angola, Malawi, Mozambique, Nepal, Sierra Leone and Zimbabwe) introducing MCV2 in 2015. Estimated global MCV2 coverage increased from 15% in 2000 to 61% in 2015 [1]”. Measles vaccine is associated with approximately 85-90% efficacy when administered routinely at 9 months of age, as recommended by WHO. This vaccination schedule reflects a compromise between declining passively-acquired maternal antibody, which may neutralize the vaccine, and the high risk of mortality and morbidity among infants due to measles disease. Efficacy increases with increasing age of vaccination, but so does the risk of unprotected infection and severe consequences in those too young for vaccination. Universal coverage with a single dose of measles vaccine at 9 months of age is usually insufficient to achieve the herd immunity threshold, and measles outbreaks will occur periodically even when countries succeed in efficient implementation of a single dose schedule.

To seroconvert primary vaccine failures following the first dose of measles-containing vaccine (MCV1), WHO, in its 2009 position paper, recommended administering a second dose of measles-containing vaccine (MCV2) in the second year of life or at least one month after the first dose [2]. Furthermore, WHO recommended that MCV2 should be included in the routine vaccination schedule in countries that had achieved at least 80% coverage of MCV1 at the national level for 3 consecutive years, as determined by the most accurate means available. Countries not meeting this criterion were encouraged to prioritize raising MCV1 coverage and implementing a high-quality periodic campaign strategy referred to as Supplementary Immunization Activities (SIAs) [3], rather than adding MCV2 to their routine schedule.

WHO also recommended that cessation of SIAs should be considered only when both MCV1 and MCV2 coverage of at least 90% had been achieved at national level for at least 3 consecutive years. Aside from current MCV2 adopters, there are few African countries which are likely to meet the former WHO coverage criterion in the near future. As of early 2016, 161 (82%) of the World’s 194 countries and territories had introduced a routine MCV2 dose into the national schedule, and MCV2 coverage stood at around 61%. Among the remaining 33 countries, 10 had achieved MCV1 coverage at or above the recommended threshold for introducing a routine MCV2 dose. Of the remaining 23 countries, 18 of them are in the African Region where, until recently, the second measles dose was provided in most countries through SIAs.

A working group of the Strategic Advisory Group of Experts (SAGE), which advises WHO on all policy decisions related to vaccines and immunization, recently undertook a series of analyses to determine if the condition for introducing a routine MCV2 dose should be retained. The group reached the following conclusions: 1) introducing a routine MCV2 dose does not impact MCV1 coverage negatively; 2) the dropout rate between MCV1 and MCV2 doses is lower in countries that met the introduction threshold for MCV2, but after 5 years dropout declines in all countries whether they met the introduction threshold or not; 3) countries with weaker immunization systems that introduced a routine MCV2 dose showed poorer performance overall than those with more robust systems; 4) using SIAs in high endemicity settings to deliver the second measles vaccine dose placed children born earlier in the inter-SIA interval at greater risk of measles infection and its consequences; 5) since routine MCV2 is administered in the second year of life, its introduction would be expected to break the barrier to receiving MCV1 after the first birthday, which is an old practice long discouraged by WHO but still evident in a number of African countries; 6) introducing a routine MCV2 dose reduces vaccine wastage where multi-dose vials are used; the resulting reduction in vaccine wastage would be expected to help overcome the hesitation that some health workers demonstrate to opening a multi-dose vial in settings where session size is small, as observed in many rural health posts; 7) for operational reasons, measles doses administered through SIAs are not always recorded on home-based records or clinic registers; the introduction of a routine MCV2 dose should lead to more accurate recording and reporting of both doses.

In October 2016 SAGE reviewed the detailed evidence presented by the working group and determined that the condition of achieving at least 80% MCV1 coverage before introducing a routine MCV2 dose should be dropped. This recommendation became WHO policy in 2016. The GAVI Alliance offers eligible countries financial support to introduce a routine MCV2 dose (and also combined measles-rubella vaccine) [4]. Since almost all of the 18 African countries currently without routine MCV2 in their national vaccination schedules are eligible for GAVI support, this is an opportune moment for those countries to apply to introduce routine MCV2 and close this small but important gap in the immunity profile of their populations.

Universal routine vaccination coverage, together with solid national commitment to invest in measles surveillance and outbreak investigation and response, holds the key to achieving measles elimination in the African Region. Measles elimination in the African Region will require a two-pronged approach: 1) strengthening of routine immunization through implementation of the Reach Every District (RED) approach, with special attention to reduction of dropout rates, 2) Supplemental Immunization Activities (SIAs) of high quality, following WHO guidelines, especially the preparedness tool.

### Implications for some African countries

Of 23 countries that have not introduced MCV2 and which do not meet the pre-2016 introduction criteria, 20 are in Africa, and 19 are in the African region of WHO Both Ethiopia and Nigeria are among those with large populations of unvaccinated children, and are now GAVI eligible for MCV2 introduction. Ethiopia and Nigeria had MCV1 coverage of 78 and 54 percent respectively in 2015, based on WHO/UNICEF estimates. Neither was eligible under the previous coverage criterion. Both are now eligible. The following discussion will focus on implications of future two dose regimes in Ethiopia and Nigeria. The literature from both countries shows that maternal knowledge of vaccines and vaccine preventable disease is among the best predictors of vaccination coverage.

### Ethiopia

The most populous country in the Horn of Africa, Ethiopia has seen slow increments in MCV1 coverage, though the most recent WHO/UNICEF estimates are still short of 80 percent. The country has a thin peripheral PHC infrastructure, strengthened by the recent deployment of thousands of female health extension workers (HEWs). The drivers of Ethiopia’s coverage are multiple, but maternal knowledge is a common factor running through most published coverage analyses. One study from Southeast Ethiopia concluded that “maternal health care utilization and knowledge of mother about vaccine and vaccine preventable disease are the main factors associated with complete immunization coverage [5]”. A study from Ambo woreda in Central Ethiopia showed that “maternal health care utilization and knowledge of mothers about the age at which the child begins and finishes vaccination are the main factors associated with complete immunization coverage [6]”. A review of data from the 2011 Demographic and Health Survey showed that “health service use and access to information on maternal and child health were found to predict full immunization coverage [7]”.

### Nigeria

The most populous country in Africa, Nigeria is home to some 160 million persons. “The available data on routine immunization in Nigeria show a disparity in coverage between Northern and Southern Nigeria, with the former performing worse. The effect of socio-cultural differences on health-seeking behaviour has been identified in the literature as the main cause of the disparity [8]”. In general, coverage is higher, and incidence of vaccine preventable diseases lower, in the south than in the north. Published articles on routine immunization in Nigeria identify several different correlates of child vaccination coverage. However, maternal knowledge figures in almost all references consulted. An analysis of determinants of routine immunization coverage in Zamfara State showed that maternal knowledge and educational status were the main determinants of immunization coverage [9]. In Osun State, mothers’ having good knowledge of immunization was a significant determinant of full immunization [10]. In rural areas of Edo State, mothers’ knowledge of immunization was significantly correlated with the rate of full immunization [11].

### The way forward in newly eligible countries

Each country introducing MCV2 needs to formulate strategies which assure high completion rates in the second year of life. In the case of Ethiopia and Nigeria, at least, assuring high levels of maternal knowledge will need to form part of those strategies. This being the case, countries which underperform in MCV1 need to make special efforts at assuring high levels of maternal knowledge. One effort to improve caregiver knowledge was undertaken in primary health care centers in Ibadan, Nigeria [12]. Four randomly selected LGAs were randomized to receive a cellphone reminder/recall only (A), a PHC Immunization Providers’ Training only (B), combined (A) and (B), or control (D). As the study endpoint, immunization completion rates were 98.6 percent for Group A, and lower figures for all other groups. Based on the performance of Group A, the authors of the Ibadan study conclude that “cellphone reminder/recall was effective in improving immunization completion in this Nigeria setting”.

Is the Ibadan study generalizable, either in Nigeria or elsewhere? Only larger scale studies will answer this question. A Cochrane review of published studies concludes that “patient reminder and recall systems in primary care settings are effective in improving immunization rates in developed countries [13]”. One study from Zimbabwe [14] and one study from Kenya [15] are confirmatory of the findings from Ibadan.

Careful tracking of MCV1 and MCV2 estimates from countries introducing two dose regimens will show the extent to which SMS reminders and other interventions for dropout reduction may serve to assure higher start and completion rates for two dose schedules. If the SAGE recommendations [16] are implemented in tandem with better sensitization of caregivers, African countries may be closer to measles elimination by 2020 than is currently the case.

## Competing interests

The authors declare no competing interest.
